# Supplementary material for: GmBTB/POZ promotes the ubiquitination and degradation of LHP1 to regulate the response of soybean to Phytophthora sojae
Source: Commun Biol. 2021 Mar 19;4:372. doi: 10.1038/s42003-021-01907-7 (PMC7979691; doi:10.1038/s42003-021-01907-7)
Supplement: Supplementary file 2 — Supplementary information [file 42003_2021_1907_MOESM2_ESM.pdf]

# GmBTB/POZ promotes the ubiquitination and degradation of LHP1 to regulate the response of soybean to *Phytophthora sojae*

**Chuanzhong Zhang[1,2†], Qun Cheng[1†], Huiyu Wang[1†], Hong Gao[1], Xin Fang[1], Xi Chen[1], Ming Zhao[1], Wanling Wei[1], Bo Song[1], Shanshan Liu[1], Junjiang Wu[3], Shuzhen Zhang[1*], Pengfei Xu[1*]**

[1]**Soybean Research Institute, Northeast Agricultural University/Key Laboratory of Soybean Biology of Chinese Education Ministry, Harbin 150030, China**

[2]**Key Laboratory of Soybean Molecular Design Breeding/Northeast Institute of Geography and Agroecology, Chinese Academy of Sciences, Harbin 150081, China**

[3]**Soybean Research Institute of Heilongjiang Academy of Agricultural Sciences/Key Laboratory of Soybean Cultivation of Ministry of Agriculture P. R. China, Harbin 150086, China**

[†]**These authors contributed equally.**

***Correspondence authors xupengfei@neau.edu.cn**

**zhangshuzhen@neau.edu.cn**

**Supplementary Figure 1 Nucleotide and amino acid sequences of GmLHP1.** The chromo domain and chromo shadow domain are shown in shadow.

```
1      AAAGGGAAAAAGGGGCGAATGGAGAAAAGCGTTAAATAAAAAACCGGAGCGATGGATCAC
61     ACTTTTCGGTATAGATGGACAACTCATCACCTCAGACTCTCGGAGAGAATGGACACAACT
121    TTTGATTTGTTGCTTCCTCCGCTCTGAAGCTGAAGCTGTTTCTGTTGTGCTAAGAAAAAA
181    AACAAAAAACAAAACAGATATAGGGAGAAAGAGAAGAAAAAAAGAACATAGTTTCTTTCA
241    GGGTTGCTCCTTTTCGATTCTTGTTGCGTTTTTGTTTGTTGTTGTTGTTGTTGTTGTTGT
301    TGTTGCTGCTGCTGCTGCAATGAAGGGTGGTGGTGGGAAGAGAAAGGCTTCTACCTCGGA
1                          M  K  G  G  G  G  K  R  K  A  S  T  S  E

361    GGCTCCGAACGATGTTGTTGTTGTTGTTGCTGCTGCTTCTGCTCCTTCTGATGATGCTGA
15       A  P  N  D  V  V  V  V  V  A  A  A  S  A  P  S  D  D  A  D

421    TTTGGGTGTTGGAGATGTCGGGGGTGTGAAAGTTCAAAACTTTGAGGGAAACGAGGGTAC
35       L  G  V  G  D  V  G  G  V  K  V  Q  N  F  E  G  N  E  G  T

481    CCAGTTGCGGAATACTGAAGAACAGGAAGAAGAACCCCACCACGTGGAGGGTGATGAAGG
55       Q  L  R  N  T  E  E  Q  E  E  E  P  H  H  V  E  G  D  E  G

541    AGGAGAAGACGAAGAAGATTATGCCGAGGGAGAGGAAGAATATGAAGAAGAAAATGGTGG
75       G  E  D  E  E  D  Y  A  E  G  E  E  E  Y  E  E  E  N  G  G

601    TGCTGTTGCTGGGGGACAACAAGGTTTTCCTGCTCTTGGTGAAAATTTCTTTGAAGTTGA
95       A  V  A  G  G  Q  Q  G  F  P  A  L  G  E  N  F  F  E  V  E
                                               Chromo domain→
661    AGCCATACGTCGTAAGAGGTTGCGCAAGGGTGAGGTGCAATACTTAATCAAATGGAATGG
115      A  I  R  R  K  R  L  R  K  G  E  V  Q  Y  L  I  K  W  N  G

721    GTGGCCCGAGACAGCCAACACTTGGGAGCCTCTGGAAAATCTAGAGTCTGTTCCTGATGT
135      W  P  E  T  A  N  T  W  E  P  L  E  N  L  E  S  V  P  D  V

781    TGTTGAAGCTTTTGAGGAGAGCTTGAAGTCCGGAAAACATCGTAAGCGCAAACGCAAGCA
155      V  E  A  F  E  E  S  L  K  S  G  K  H  R  K  R  K  R  K  H
                                      ←Chromo domain
841    TGTAGTGCATCATACTCAACCCAAGAAGCGGCTGGAGCGTTCGACAACTCCTTACAGCCT
175      V  V  H  H  T  Q  P  K  K  R  L  E  R  S  T  T  P  Y  S  L

901    TCGGCGTTTTTCAACTAGTACAGCTGAGAACCATACACAGTCTGCTCCTCCTCTTAATGA
195      R  R  F  S  T  S  T  A  E  N  H  T  Q  S  A  P  P  L  N  D

961    TCCTAGCCTTCCAGATATTCCCGCCTTCCCTCACACTGTGCTGTTTTCTGATGATGTGGG
215      P  S  L  P  D  I  P  A  F  P  H  T  V  L  F  S  D  D  V  G

1021   AAATGGTGCTGAAGGCAGCAGTCTTAGAAAAGCCACACCCAGTAATGCTAACAGGTCTGC
235      N  G  A  E  G  S  S  L  R  K  A  T  P  S  N  A  N  R  S  A

1081   AAATGGTTCAGAACAAAATATTAAAAGGAATGAGGAAAATGATTATGATCCTGTGCTTAG
255      N  G  S  E  Q  N  I  K  R  N  E  E  N  D  Y  D  P  V  L  S

1141   TGAGCTTAAAGCTATGACTTCTAATGGGAATGATGCAGACAGGCTTGCAATACGGATTCC
275      E  L  K  A  M  T  S  N  G  N  D  A  D  R  L  A  I  R  I  P

1201   AGAAGCTAAGGGTTCTGGGCCTTCTGGCAGTAATGGTCAAATGGATGCTAAATCAAAGGG
295      E  A  K  G  S  G  P  S  G  S  N  G  Q  M  D  A  K  S  K  G

1261   GGCTTGCATGGAAACAAGTGAAAGTGACCGCTGCCGGGGATCCAAAAGGAGAAAATGTGG
315      A  C  M  E  T  S  E  S  D  R  C  R  G  S  K  R  R  K  C  G

1321   TTCCGTAAAGAGGTTCAAGAAAGAACTATATGCTAATGAGCCTGCTAATGCGGAAAATCC
335      S  V  K  R  F  K  K  E  L  Y  A  N  E  P  A  N  A  E  N  P

1381   AGTTAGCATGCCTGTTGGTACAGCTGAGCCAGAACGAACAAGAGATGCTGGTAGTGGTGG
355      V  S  M  P  V  G  T  A  E  P  E  R  T  R  D  A  G  S  G  G
                                        Chromo shadow domain→
1441   CAATACTAATCATGCCAGACCTGCTAGCAATATTGTAAACATTGTAAAGCCAGTAGGCTA
375      N  T  N  H  A  R  P  A  S  N  I  V  N  I  V  K  P  V  G  Y

1501   TTCAGCTTCAGTAGCCAGTGGAATGCAGGATGTTTTAGTAACCTTTGTGGCTTCAAAGTC
395      S  A  S  V  A  S  G  M  Q  D  V  L  V  T  F  V  A  S  K  S

1561   TGATGGAACAGAAGTGATGGTTGACAACAAGTATCTAAAAGCATTTAATCCACTTCTTCT
415      D  G  T  E  V  M  V  D  N  K  Y  L  K  A  F  N  P  L  L  L

1621   GATCAATTTCTATGAGCAGCATCTTCGCTACAGCCCTACATTGTGACCAAACAGAGAATA
435      I  N  F  Y  E  Q  H  L  R  Y  S  P  T  L  *
                    ←Chromo shadow domain
1681   GAAGGTGCTTTTGAGTTTGTAGTTAGGTTCACACGATTGTGCTTAGTTAGTAGTTTTGTT
1741   GAAGAATGTCTTACTTTTATAGATGCAGAAGATAGTAAAGGGTAAGAAATGCATTGTTTG
1801   TTAGTTACACGTAGGTATGATTTTAACAAGTGGTTGTCAGTGTGGCTATATACTGACTGA
1861   TGCATGCTGATGGCATAAATAACTATATATTTGAATGCCTCAAACACACGAGGATAGACA
1921   ATAGTTTCTTAGGAGTTAGTCAAGAATGAAAAACTATTTTGGATTATTGTAAATTTTTAT
1981   GGAAGTTCTTGGCTATTTCTGAGCGATTTGTTTGCCTATTATAATTTGAATACTGATTGG
2041   TTGATTGATTGACATGATTCTAAAGGAAAATCCACAATTAACTGTTAA
```

**Supplementary Figure 2 Detection of transgenic soybean lines in this study. (a)** Immunoblots showing the expression of GmBTB/POZ-Myc fusion protein in three independent T4 *GmBTB/POZ-OE* soybean plants and the WT controls. The total protein extracts were analyzed in a 12% SDS-PAGE gel for the immunoblot probed with anti-Myc antibody. **(b)** Relative *GmBTB/POZ* expression levels in T4 *GmBTB/POZ-OE* soybean plants. **(c)** The three independent T4 *GmBTB/POZ-RNAi* and WT soybean plants were tested using Quickstix Kit for Liberty Link bar strips. **(d)** Relative *GmBTB/POZ* expression levels in T4 *GmBTB/POZ-RNAi* soybean plants. **(e)** Immunoblots showing the expression of GmLHP1-Flag fusion protein in three independent T4 *GmLHP1OE* soybean plants and the WT controls. The total protein extracts were analyzed in a 12% SDS-PAGE gel for the immunoblot probed with anti-Flag antibody. **(f)** Relative *GmLHP1* expression levels in T4 *GmLHP1OE* soybean plants. **(g)** Southern blot analysis of T4 *GmLHP1RNAi* and WT soybean plants. Twenty micrograms of genomic DNA digested by the restriction enzyme Hind Ⅲ was hybridized with the probe derived from the bar-specific fragment (354 bp). **(h)** Relative *GmLHP1* expression levels in T4 *GmLHP1RNAi* soybean plants. **(i)** Immunoblots showing the expression of (domain+C)-Myc fusion protein in three independent *(domain+C)-OE* soybean hairy roots and the EV controls. The total protein extracts were analyzed in a 12% SDS-PAGE gel and probed with an anti- Myc antibody for the immunoblot. **(j)** Immunoblots showing the expression of (N+domain)-Myc fusion protein in three independent *(N+domain)-OE* soybean hairy roots and the EV controls. The total protein extracts were analyzed in a 12% SDS-PAGE gel and probed with an anti-Myc antibody for the immunoblot. **(k)** Immunoblots showing the expression of GmWRKY40-Flag fusion protein in three independent *GmWRKY40-OE* soybean hairy roots. The total protein extracts were analyzed in a 12% SDS-PAGE gel and probed with an anti-Flag antibody for the immunoblot. **(l)** The *GmWRKY40-RNAi* transgenic soybean hairy roots were tested using Quickstix Kit for Liberty Link bar strips. **(m)** Immunoblots showing the expression of GmLHP1-8-Flag fusion protein in three independent *GmLHP1-8-OE* soybean hairy roots and the EV controls. The total protein extracts were analyzed in a 12% SDS-PAGE gel and probed with an anti-Flag antibody for the immunoblot.

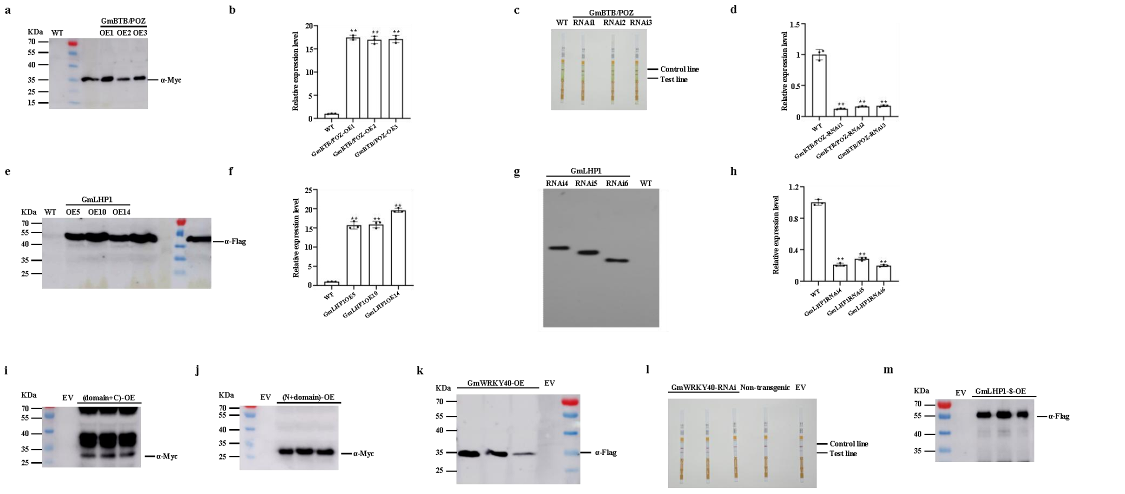

**Supplementary Figure 3 The active region of GmBTB/POZ involved in interaction and ubiquitination of GmLHP1. (a)** Scheme of GmBTB/POZ different regions inserted into yeast vectors pGBD. **(b)** GmLHP1 interacts with GmBTB/POZ different regions in yeast cells. The yeast cells were selected on SD medium lacking Leu and Trp (DDO), and interaction was assessed based on their ability to grow on selective SD medium lacking Leu, Trp, His, and Ade (QDO) or SD medium lacking Leu, Trp, His, and Ade (QDO) but containing X-α-Gal for 3 days at 30°C. **(c)** BiFC analysis of the interaction of GmLHP1 with GmBTB/POZ different regions. The bright-field, YFP fluorescence (yellow), chlorophyll autofluorescence (red), and combined images were visualized under a confocal microscope at 16 h after transfection. Scale bars indicate 10 μm. **(d)** *In vitro* cell-free degradation assays of GmLHP1-His in protein extracts from *(domain+C)-OE* and *(N+domain)-OE* transgenic soybean hairy roots. **(e)** Scheme of GmBTB/POZ (domain+C) and (N+domain) regions inserted into yeast vectors pGBD. **(f)** GmLHP1 interacts with the (domain+C) and (N+domain) regions of GmBTB/POZ in yeast cells. **(g)** BiFC analysis of the interaction of GmLHP1 with GmBTB/POZ (domain+C) and (N+domain) regions. **(h)** The (domain+C) and (N+domain) of GmBTB/POZ promotes the ubiquitination of GmLHP1 *in vivo*. GmLHP1-Flag was immunoprecipitated using anti-Flag-Tag Mouse mAb (Agarose Conjugated) from *GmLHP1-OE*, *GmLHP1-OE/(domain+C)-OE* and *GmLHP1-OE/(N+domain)-OE* transgenic soybean hairy roots by high-efficiency *A. rhizogenes*-mediated transformation. The transgenic hairy roots were treated with 100 μM MG132 for 8 h before extraction. The immunoprecipitated protein was examined using anti-Flag and anti-ubi antibodies.

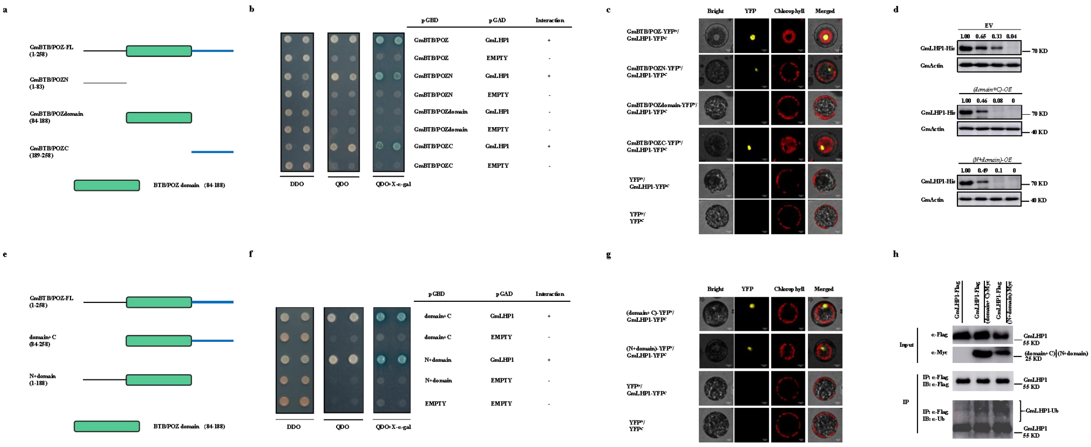

a
GmBTB/POZ-FL
(1-258)
GmBTB/POZN
(1-83)
GmBTB/POZdomain
(84-188)
GmBTB/POZC
(189-258)
BTB/POZ domain (84-188)
b
pGBD
pGAD
Interaction
GmBTB/POZ
GmLHP1
+
GmBTB/POZ
EMPTY
-
GmBTB/POZN
GmLHP1
+
GmBTB/POZN
EMPTY
-
GmBTB/POZdomain
GmLHP1
-
GmBTB/POZdomain
EMPTY
-
GmBTB/POZC
GmLHP1
+
GmBTB/POZC
EMPTY
-
DDO
QDO
QDO+X-α-gal
c
Bright
YFP
Chlorophyll
Merged
GmBTB/POZ-YFPN/
GmLHP1-YFPC
GmBTB/POZN-YFPN/
GmLHP1-YFPC
GmBTB/POZdomain-YFPN/
GmLHP1-YFPC
GmBTB/POZC-YFPN/
GmLHP1-YFPC
YFPN/
GmLHP1-YFPC
YFPN/
YFPC
d
EV
1.00 0.65 0.33 0.04
GmLHP1-His
70 KD
GmActin
(domain+C)-OE
1.00 0.46 0.08 0
GmLHP1-His
70 KD
GmActin
40 KD
(N+domain)-OE
1.00 0.49 0.1 0
GmLHP1-His
70 KD
GmActin
40 KD
e
GmBTB/POZ-FL
(1-258)
domain+C
(84-258)
N+domain
(1-188)
BTB/POZ domain (84-188)
f
pGBD
pGAD
Interaction
domain+C
GmLHP1
+
domain+C
EMPTY
-
N+domain
GmLHP1
+
N+domain
EMPTY
-
EMPTY
EMPTY
-
DDO
QDO
QDO+X-α-gal
g
Bright
YFP
Chlorophyll
Merged
(domain+C)-YFPN/
GmLHP1-YFPC
(N+domain)-YFPN/
GmLHP1-YFPC
YFPN/
GmLHP1-YFPC
YFPN/
YFPC
h
GmLHP1-Flag
GmLHP1-Flag
(domain+C)-Myc
GmLHP1-Flag
(N+domain)-Myc
Input
α-Flag
GmLHP1
55 KD
α-Myc
(domain+C)|(N+domain)
25 KD
IP
IP: α-Flag
IB: α-Flag
GmLHP1
55 KD
IP: α-Flag
IB: α-Ub
GmLHP1-Ub
GmLHP1
55 KD

**Supplementary Figure 4 Subcellular localization and transcriptional activation analysis of GmLHP1. (a)** Subcellular localization analysis of GmLHP1. **(b)** Transcriptional activation analysis of GmLHP1.

a

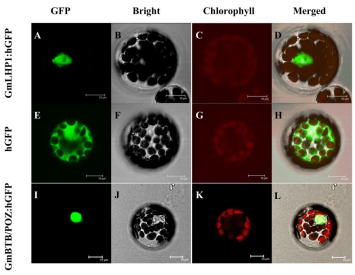


b

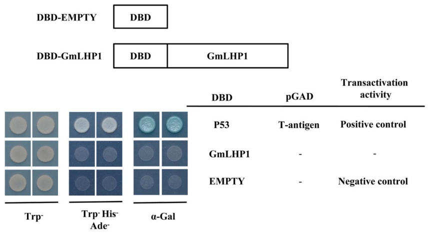

**Supplementary Figure 5 Transcriptomic analysis of gene expression profiles in response to *GmLHP1* overexpression. (a)** Volcano plots of significantly differentially expressed genes in GmLHP1OE vs. wild-type soybean plants after RNA-seq analysis. **(b)** GO functional classification of differentially expressed genes. The differentially expressed genes were placed into the three main GO categories: biological process, cellular component, and molecular function.

**a**

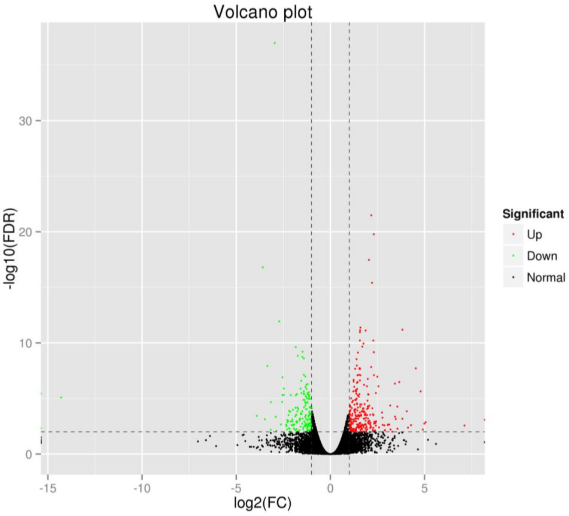


GmLHP1OE VS WT

**b**

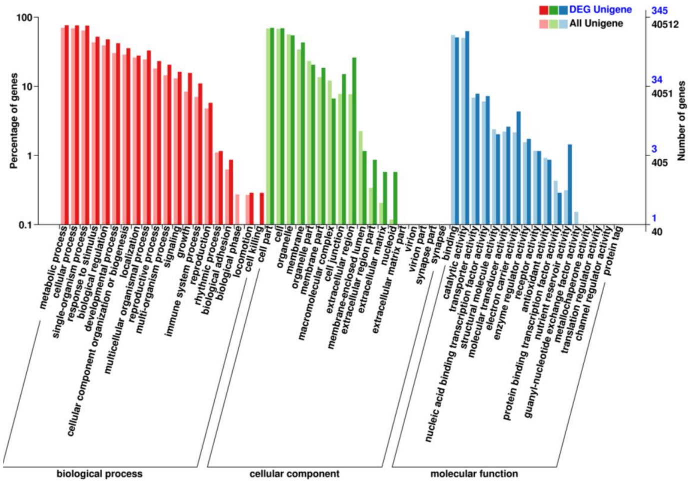

**Supplementary Figure 6 Expression kinetics of *GmBTB/POZ*, *GmLHP1*, *GmWRKY40*, and *GmPR1* in response to *P. sojae* infection in resistant soybean cultivar 'Suinong 10'.** The relative expression levels are expressed as fold changes relative to the transcript level of each gene at the 0 h time point. Fourteen-day-old soybean plants were used for analysis. The housekeeping gene *GmEF1* was used as an internal control to normalize the data.

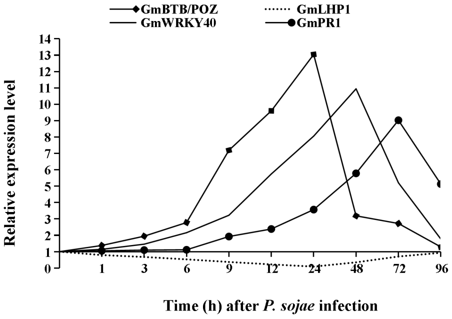

**Supplementary Figure 7 Nucleotide and amino acid sequences of GmWRKY40.** The WRKY domain is shown in shadow.

```
1       AAACAAGTAGCGGATATATTCGCATGTGTGTCTTTTTCCTCACTCTCTTTCCTCTCAGTT
61      TCCTTCTTCAACTTGTGCTCCTTCTAAGTGCTACTCCCTCCCTATATATATATGCTTCTC
1                                                            M  L  L

121     TCACTTCACAAACTTAATTTGAACCTCTCACCTTGCTCATCATCTGGAATTTATTTGGCA
4        S  L  H  K  L  N  L  N  L  S  P  C  S  S  S  G  I  Y  L  A

181     GTCATGGATTATTCATCATGGATTAACACTTCCTTGGATCTCAACATTAATCTCCATAGG
24       V  M  D  Y  S  S  W  I  N  T  S  L  D  L  N  I  N  L  H  R

241     GTTCACGAAGAACTTCCCAAAAAGCAGGTGGAAAACAATTTTTTCTCATTGGATTTGGAG
44       V  H  E  E  L  P  K  K  Q  V  E  N  N  F  F  S  L  D  L  E

301     GTGAAGAAATCTTCTGTAAAACAAGAGTCAGCAGGTGCCTTGGCGGAGGAACTGAAGCGG
64       V  K  K  S  S  V  K  Q  E  S  A  G  A  L  A  E  E  L  K  R

361     GTGAGTGCAGAAAACAAGAAGTTAACCGAAATGCTCACAGAGATGTGTGAGAACTACAAC
84       V  S  A  E  N  K  K  L  T  E  M  L  T  E  M  C  E  N  Y  N

421     ACTTTGCGAAGCAATTTGATGGAATACATGAGGAAGAATCCTGATAAGGAGCTCAGCTCA
104      T  L  R  S  N  L  M  E  Y  M  R  K  N  P  D  K  E  L  S  S

481     TCAAGGAAAAGGAAGTCTGCAAGCAGCAACAACAATAGTACTATTCCAATGGGAGTCAAC
124      S  R  K  R  K  S  A  S  S  N  N  N  S  T  I  P  M  G  V  N

541     GGAACCTCTGAAAGCAGCTCAACCGATGAGGAATCCTGCAAGAATCCAAAGGAAGACATG
144      G  T  S  E  S  S  S  T  D  E  E  S  C  K  N  P  K  E  D  M

601     AAGACAAAAATTTCAAGAGTTTATATGAGGACAGAAGCATCTGATACAAGCCTTATTGTG
164      K  T  K  I  S  R  V  Y  M  R  T  E  A  S  D  T  S  L  I  V
                                                 WRKY domain→
661     AAAGATGGATATCAATGGAGGAAATATGGACAAAAGGTGACCAGAGATAACCCTTCTCCA
184      K  D  G  Y  Q  W  R  K  Y  G  Q  K  V  T  R  D  N  P  S  P

721     AGAGCATATTTCAAGTGCTCTTTTGCTCCAAGCTGCCCGGTCAAAAAGAAGGTGCAAAGA
204      R  A  Y  F  K  C  S  F  A  P  S  C  P  V  K  K  K  V  Q  R

781     AGTGTGGATGATCAATCTGTTCTGGTTGCAACTTATGAAGGAGAGCACAATCATACACAC
224      S  V  D  D  Q  S  V  L  V  A  T  Y  E  G  E  H  N  H  T  H

841     CCTTCTCAAATGGAGGTAACAACAGGTTCCAACCGTTCAGTATCATGTTCAGCGTCTCTT
244      P  S  Q  M  E  V  T  T  G  S  N  R  S  V  S  C  S  A  S  L
        ←WRKY domain
901     AGCTCTTCCGCACCAACAGTTACCCTTGACTGGACAAAATCCAAGTCCAGCAGTGAGTCT
264      S  S  S  A  P  T  V  T  L  D  W  T  K  S  K  S  S  S  E  S

961     AAGAATGTAAATCCCAAAACTGAATCACCAGAAGTCCCGCAGGTTTTGGTAGAACAGATG
284      K  N  V  N  P  K  T  E  S  P  E  V  P  Q  V  L  V  E  Q  M

1021    GCTACTTCCTTGACCAAGGATCCTAATTTCAGAGCAGCACTAGTTGCCGCCATCTCTGGA
304      A  T  S  L  T  K  D  P  N  F  R  A  A  L  V  A  A  I  S  G

1081    AAAATGTTGCACAATAATAATTAAGTAGAAAATGAGTCAACTGTATTTAACTCATTGTTA
324      K  M  L  H  N  N  N  *

1141    AGCAGTATTCTATAAGTGGATCTTGTTTAGAGCACATGATCTCTTTGTAAATACAACATA
1201    GGACATTGCTACGTATGTAGAGCTCCTCAATTTAATCAATCCTTTTTGGTGGAATAGAGC
1261    TCCACTCAGGATCGATAGCATGTCTAGCTGATTGTGCATTTGCTAATCATGCTCATCTCA
1321    GTCTTCTCTATTCTTCTGCATCTGGATGATTAAAATTAGGTTTACAATTAAGTTCTGGCG
1381    AAGTTTCTATAGCATACAAAAGCAAAGAGAAACTTTGTTTCGAAAAGTGCAACTCTATTG
1441    AGTATTGAGTATTGACAATACAATATCTCCTTGTCATTTTCCAACTGTCCATTTGGAACA
1501    CCGCTTCTCCCTTTTTATCTCACAAAAGATTAATAATAGGGATGCAACGATCGCAACTTC
1561    TCAGGCTGTTTAATACATTATATAGATTCAGACTCCTAGTCTGAGATTTTTCACTTCAC
```

**Supplementary Figure 8 The early-flowering phenotype of *GmLHP1RNAi* soybean plants (43-d-old plants) under artificial long-day condititions.**

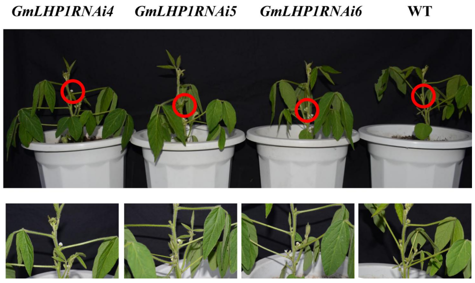

# Supplementary Figure 9

Figure 2a

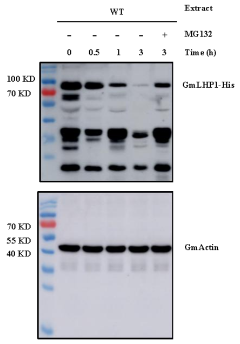


Figure 2c

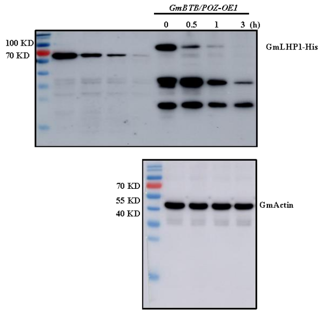


Figure 2d

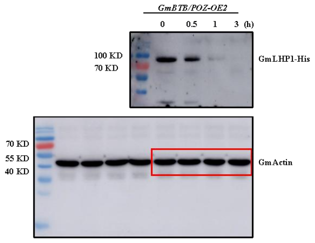


Figure 2e

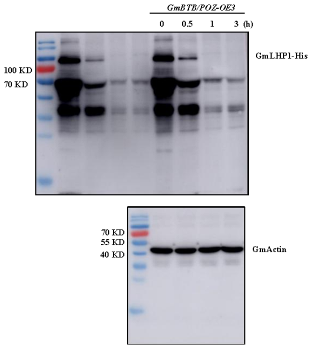


Figure 2f

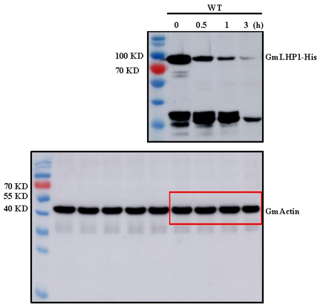


Figure 2g

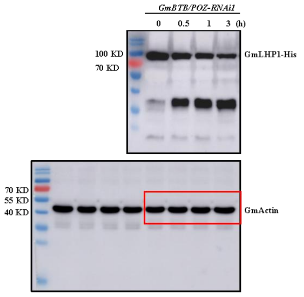


Figure 2h

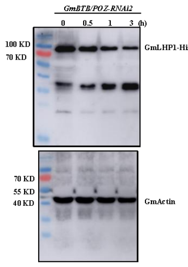


Figure 2i

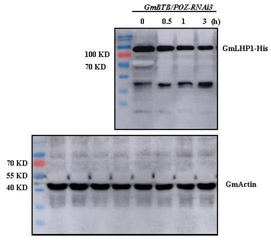


Figure 2k

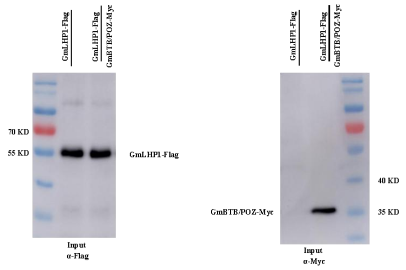


Figure 2k

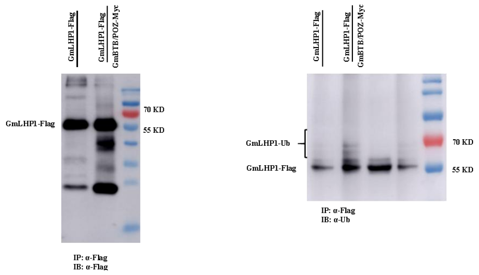


Figure 7g

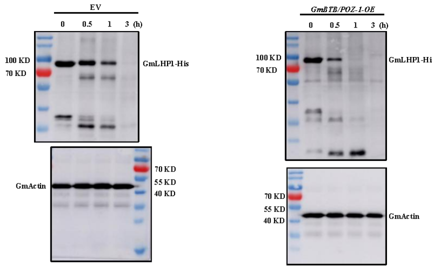

Figure 7h

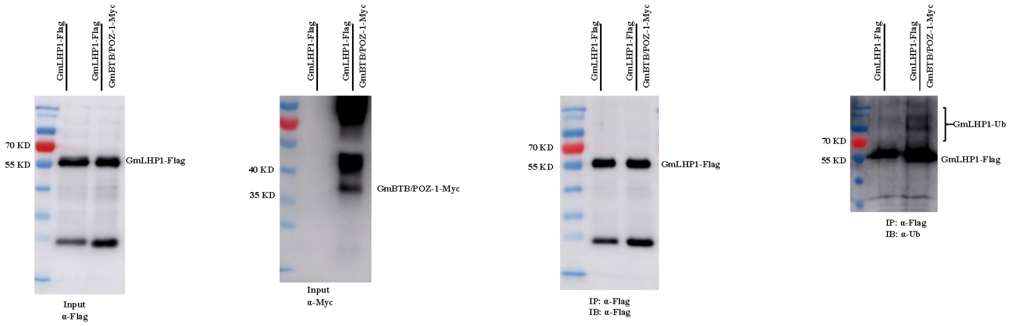
GmLHP1-Flag
GmLHP1-Flag
GmBTB/POZ-1-Myc
70 KD
55 KD
GmLHP1-Flag
Input
α-Flag
40 KD
35 KD
GmBTB/POZ-1-Myc
Input
α-Myc
70 KD
55 KD
GmLHP1-Flag
IP: α-Flag
IB: α-Flag
GmLHP1-Ub
70 KD
55 KD
GmLHP1-Flag
IP: α-Flag
IB: α-Ub

**Supplemental Table 1. List of primers used in this study.**

**Primers for quantitative real-time PCR**

| Primer name | Primers (5'-3') |
|---|---|
| *GmLHP1-qPCR* | F: AAGGTTTTCCTGCTCTTGGTGA |
| | R: CCGCTTCTTGGGTTGAGTATG |
| *GmPR1-qPCR* | F: TGAAAATGTGGGTTGATGAGAAAT |
| | R: AAGTGATGAAAGTGCCTCCGTT |
| *GmMEKK2-qPCR* | F: CGGGAATACACGAGGGACAT |
| | R: GCACATCCCAAAGCCCAT |
| *GmWRKY40-qPCR* | F: GAGGAACTGAAGCGGGTGA |
| | R: AGGTTCCGTTGACTCCCATT |
| *GmCRK2-qPCR* | F: GCGGCTCAATCAACCAGTG |
| | R: GTGGAGCAGTTGCGGATA |
| *GmNAC90-qPCR* | F: GCCATAGACAGAGTTATCCCAGTTA |
| | R: CCTGTTGCTTTCCAATACCCA |
| *GmNAC29-qPCR* | F: ACCAAGCCACATCAAGACCAT |
| | R: ATACCCAGACACGGTTGCTC |
| *GmERF104-qPCR* | F: AGCCTCCAAGGTCCTCATCA |
| | R: CTATTAGGGTCACGGATTTCTGC |
| *GmbHLH35-qPCR* | F: AACGATAGGCTTTTGGCACTTA |
| | R: CAGAATAGGGAGGTCCTGGTCA |
| *GmMYB70-qPCR* | F: AGGGCTCACGCCAGGTTC |
| | R: CGATGGGCTGCCAGGGTT |
| *GmMLP34-qPCR* | F: TAAAGGTGAATGGGGCAAAGA |
| | R ATGAGCGTCAGGAATGTGGTC |
| *GmBTB/POZ-qPCR* | F: CTCCGACGACGCTCCCAT |
| | R: GGTGCCACTCCGCCTTTC |
| *GmEF1-qPCR* | F: CCACTGCTGAAGAAGATGATGATG |
| | R: AAGGACAGAAGACTTGCCACTC |
| *TEF1-qPCR* | F: TGATCGTGCTGAACCACCC |
| | R: CGAGCGACGGTCCATCTT |
| *GmLHP1-8-qPCR* | F: AAGGTTTTCCTGCTCTTGGTGA |
| | R: AAAAACGCCGAAGGCTGTAA |
| *GmBTB/POZ-1* | F: CTCCGACGACGCTCCCAT |
| *-qPCR* | R: GGTGCCACTCCGCCTTTC |

**Primers for constructs in soybean genetic transformation**

| Primer name | Primers (5'-3') |
|---|---|
| *GmLHP1-Flag* | F:ctcttgacccatggtaGATTACAAGGATGACGACGATAAGATGAAGGG TGGTGGTGGG |
| | R: gtcacctgtaattcacacgtgTCACAATGTAGGGCTGTAGC |
| *GmLHP1RNAi1* | F: CCGCTCGAGGATCCAAAAGGAGAAAATGTG |
| | R: CATGCCATGGCAATGTAGGGCTGTAGCGAA |
| *GmLHP1RNAi2* | F: GCTCTAGAGATCCAAAAGGAGAAAATGTG |

| | |
|---|---|
| | R: CGGGATCCCAATGTAGGGCTGTAGCGAA |
| *GmBTB/POZ-Myc* | F: CCATGGTGATGCCTCCGCGGCGGTGCAC<br>R: CACGTGAAATGCGCCTGCAGTATTTA |
| *GmBTB/POZRNAi1* | F: CCGCTCGAGGAGCGTGAGAGCACAG<br>R: CATGCCATGGCTCGTTGGCCTCCTGG |
| *GmBTB/POZRNAi2* | F: GCTCTAGAGAGCGTGAGAGCACAGC<br>R: CGGGATCCCTCGTTGGCCTCCTGGTA |

**Primers for constructs in transformation of soybean hairy roots**

| **Primer name** | **Primers (5’-3’)** |
|---|---|
| *GmLHP1-Flag* | F:CTCGAGATGGATTACAAGGATGACGACGATAAGATGAAGGG TGGTGGTGGGA<br>R: CTCGAGTCACAATGTAGGGCTGTAGCGA |
| *GmLHP1RNAi1* | F: CCGCTCGAGGATCCAAAAGGAGAAAATGTG<br>R: CATGCCATGGCAATGTAGGGCTGTAGCGAA |
| *GmLHP1RNAi2* | F: GCTCTAGAGATCCAAAAGGAGAAAATGTG<br>R: CGGGATCCCAATGTAGGGCTGTAGCGAA |
| *GmBTB/POZ-Myc* | F: CCATGGTGATGCCTCCGCGGCGGTGCAC<br>R: CACGTGAAATGCGCCTGCAGTATTTA |
| *GmBTB/POZRNAi1* | F: CCGCTCGAGGAGCGTGAGAGCACAG<br>R: CATGCCATGGCTCGTTGGCCTCCTGG |
| *GmBTB/POZRNAi2* | F: GCTCTAGAGAGCGTGAGAGCACAGC<br>R: CGGGATCCCTCGTTGGCCTCCTGGTA |
| *GmLHP1-Flag+GmBTB/POZ-Myc2* | F:CTCGAGATGGATTACAAGGATGACGACGATAAGATGAAGGG TGGTGGTGGGA<br>R: CTCGAGTCACAATGTAGGGCTGTAGCGA |
| *GmWRKY40-Flag* | F:caaatctatctctctcgagATGGATTACAAGGATGACGACGATAAGATG CTTCTCTCACTTCACAAACTT<br>R: attattatggagaaactcgagTTAATTATTATTGTGCAACATTTTTCCA |
| *GmWRKY40RNAi1* | F: CCGCTCGAGTAACCGAAATGCTCACAGA<br>R: CATGCCATGGGTTGGAACCTGTTGTTACCTC |
| *GmWRKY40RNAi2* | F: GCTCTAGATAACCGAAATGCTCACAGA<br>R: CGGGATCCGTTGGAACCTGTTGTTACCTC |
| *(domain+C)-Myc* | F: ctcttgaccatggtgagatctGCTTCCACCGCCGATGTC<br>R: accgttaattaaccccacgtgAAATGCGCCTGCAGTATTTAATAG |
| *GmLHP1-Flag+ (domain+C)-Myc2* | F:CTCGAGATGGATTACAAGGATGACGACGATAAGATGAAGGG TGGTGGTGGGA<br>R: CTCGAGTCACAATGTAGGGCTGTAGCGA |
| *(N+domain)-Myc* | F: ctcttgaccatggtgagatctATGCCTCCGCGGCGGTGC<br>R: accgttaattaaccccacgtgCATTTTGGCGATCAAATACTTTTC |
| *GmLHP1-Flag+ (N+domain)-Myc2* | F:CTCGAGATGGATTACAAGGATGACGACGATAAGATGAAGGG TGGTGGTGGGA<br>R: CTCGAGTCACAATGTAGGGCTGTAGCGA |
| *GmBTB/POZ-1-Myc* | F: ctcttgaccatggtgagatctATGCCTCCGCGGCGGTGC |

| | |
|---|---|
| | R: accgttaattaaccccacgtgTGTTTCATATATTTCCACAACAAGACG |
| *GmLHP1-Flag+* | F:CTCGAGATGGATTACAAGGATGACGACGATAAGATGAAGGG |
| *GmBTB/POZ-1-Myc2* | TGGTGGTGGGA |
| | R: CTCGAGTCACAATGTAGGGCTGTAGCGA |
| *GmLHP1-8-Flag* | F:CTCGAGATGGATTACAAGGATGACGACGATAAGATGAAGGG |
| | TGGTGGTGGGA |
| | R: CTCGAGTCACAATGTAGGGCTGTAGCGA |

## Primers for constructs in yeast two-hybrid assays

| Primer name | Primers (5'-3') |
|---|---|
| *GmLHP1-BD* | F: GAATTCATGAAGGGTGGTGGTGGGA |
| | R: GTCGACTCACAATGTAGGGCTGTAGCGA |
| *GmLHP1-AD* | F: GAATTCATGAAGGGTGGTGGTGGGAA |
| | R: GAGCTCTCACAATGTAGGGCTGTAGCGA |
| *GmBTB/POZ-BD* | F: atggccatggaggccgaattcAATTGGGATAAAGCTATTTCTAACTATGC |
| | R: ccgctgcaggtcgacggatccAAATGCGCCTGCAGTATTTAATAG |
| *GmBTB/POZ-AD* | F: gccatggaggccagtgaattcAATTGGGATAAAGCTATTTCTAACTATGC |
| | R: cagctcgagctcgatggatccAAATGCGCCTGCAGTATTTAATAG |
| *GmBTB/POZN* | F: atggccatggaggccgaattcATGCCTCCGCGGCGGTGC |
| *-BD* | R: ccgctgcaggtcgacggatccGTTAAGATTGGGAGAGGGGAGAG |
| *GmBTB/POZ* | F: atggccatggaggccgaattcGCTTCCACCGCCGATGTC |
| *domain –BD* | R: ccgctgcaggtcgacggatccCATTTTGGCGATCAAATACTTTTC |
| *GmBTB/POZC* | F: atggccatggaggccgaattcAATTGGGATAAAGCTATTTCTAACTATGC |
| *–BD* | R: ccgctgcaggtcgacggatccAAATGCGCCTGCAGTATTTAATAG |
| *(domain+C)-BD* | F: atggccatggaggccgaattcATGGCTTCCACCGCCGAT |
| | R: ccgctgcaggtcgacggatccCTAAAATGCGCCTGCAGTATTTAA |
| *(N+domain)-BD* | F: atggccatggaggccgaattcATGCCTCCGCGGCGGTGC |
| | R: ccgctgcaggtcgacggatccCTACATTTTGGCGATCAAATACTTTT |

## Primers for constructs in pull-down assays

| Primer name | Primers (5'-3') |
|---|---|
| *GmLHP1-His* | F: GGAATTCAATGAAGGGTGGTGGTGG |
| | R: GGTCGACCAATGTAGGGCTGTAGCGAA |
| *GmBTB/POZ-GST* | F: ccgcgtggatccccggaattcATGCCTCCGCGGCGGTGC |
| | R: gtcacgatgcggccgctcgagCTAAAATGCGCCTGCAGTATTTAA |

## Primers for constructs in LCI assays

| Primer name | Primers (5'-3') |
|---|---|
| *GmLHP1-nLUC* | F: gagctcggtacctccggatccATGAAGGGTGGTGGTGGGA |
| | R: gatctggtcgactccactagtCAATGTAGGGCTGTAGCGAAGA |
| *GmBTB/POZ-ccLUC* | F: cggggcggtacctccggatccATGCCTCCGCGGCGGTGC |
| | R: ctgcaggtcgactccactagtCTAAAATGCGCCTGCAGTATTTAA |

**Primers for constructs in BiFC assays**

| Primer name | Primers (5’-3’) |
|---|---|
| *GmBTB/POZ-NYFP* | F: GGAATTCGAGCGTGAGAGCACAGCA |
| | R: GGGTACCAAAATGCGCCTGCAGTAT |
| *GmLHP1-CYFP* | F: GAATTCATGAAGGGTGGTGGTGGG |
| | R: CGACACAATGTAGGGCTGTAGCGAA |
| *GmBTB/POZN* | F: CTCGAGCATGCCTCCGCGGCGGTGC |
| *-NYFP* | R: GAATTCGTTAAGATTGGGAGAGGGGAG |
| *GmBTB/POZ* | F: CTCGAGCATGGCTTCCACCGCCGAT |
| *domain-NYFP* | R: GAATTCCATTTTGGCGATCAAATACTTTT |
| *GmBTB/POZC* | F: CTCGAGCATGAATTGGGATAAAGCTATTTCTAACT |
| *-NYFP* | R: GAATTCAAATGCGCCTGCAGTATTTAA |
| *(domain+C)* | F: CTCGAGCATGGCTTCCACCGCCGAT |
| *-NYFP* | R: GAATTCAAATGCGCCTGCAGTATTTAA |
| *(N+domain)* | F: CTCGAGCATGCCTCCGCGGCGGTGC |
| *-NYFP* | R: GAATTCCATTTTGGCGATCAAATACTTTT |

**Primers for subcellular localization**

| Primer name | Primers (5’-3’) |
|---|---|
| *GmLHP1-1302* | F: catctagtcatgccatggca ATGAAGGGTGGTGGTGGGA |
| | R: cgacctcgaggaagatct AATGTAGGGCTGTAGCGAAGATGC |
| *GmLHP1-1-1302* | F: actcttgaccatggtagatctgGAAAATTTCTTTGAAGTTGAAGCCA |
| | R: aagttcttctcctttactagtCAATGTAGGGCTGTAGCGAAGA |
| *GmLHP1-2-1302* | F: actcttgaccatggtagatctgATGAAGGGTGGTGGTGGGA |
| | R: aagttcttctcctttactagtACCACTACCAGCATCTCTTGTTCG |
| *GmLHP1-3-1302* | F: actcttgaccatggtagatctgAAGAAAGAACTATATGCTAATGAGCCTG |
| | R: aagttcttctcctttactagtCAATGTAGGGCTGTAGCGAAGA |
| *GmLHP1-4-1302* | F: actcttgaccatggtagatctgATGAAGGGTGGTGGTGGGA |
| | R: aagttcttctcctttactagtGTCACTTTCACTTGTTTCCATGCA |
| *GmLHP1-5-1302* | F: actcttgaccatggtagatctgACAACTCCTTACAGCCTTCGGC |
| | R: aagttcttctcctttactagtCAATGTAGGGCTGTAGCGAAGA |
| *GmLHP1-6-1302* | F: actcttgaccatggtagatctgAAACGCAAGCATGTAGTGCATC |
| | R: aagttcttctcctttactagtCAATGTAGGGCTGTAGCGAAGA |
| *GmLHP1-7-1302* | F: actcttgaccatggtagatctgATGAAGGGTGGTGGTGGGA |
| | R: aagttcttctcctttactagtCTCCTCAAAAGCTTCAACAACATC |
| *GmLHP1-8-1302* | F: actcttgaccatggtagatctgATGAAGGGTGGTGGTGGGA |
| | R: aagttcttctcctttactagtCAATGTAGGGCTGTAGCGAAGA |
| *GmBTB/POZ* | F: CCATGGTTATGCCTCCGCGGCGGTGC |
| *-1302* | R: ACTAGTAAATGCGCCTGCAGTATTTAAT |
| *GmBTB/POZ* | F: actcttgaccatggtagatctgATGCCTCCGCGGCGGTGC |
| *-1-1302* | R: aagttcttctcctttactagtTGTTTCATATATTTCCACAACAAGACG |
| *GmBTB/POZ* | F: actcttgaccatggtagatctgATGCCTCCGCGGCGGTGC |
| *-2-1302* | R: aagttcttctcctttactagtATAGTTAGAAATAGCTTTATCCCAATTCA |
| *GmBTB/POZ* | F: actcttgaccatggtagatctgATGCCTCCGCGGCGGTGC |

| | |
|---|---|
| *-3-1302* | R: aagttcttctcctttactagtCATTTTGGCGATCAAATACTTTTC |
| *GmBTB/POZ* | F: actcttgaccatggtagatctgATGCCTCCGCGGCGGTGC |
| *-4-1302* | R: aagttcttctcctttactagtATTATCAAGGGACGCTTCAGCA |
| *GmBTB/POZ* | F: actcttgaccatggtagatctgGCTTCCACCGCCGATGTC |
| *-5-1302* | R: aagttcttctcctttactagtAAATGCGCCTGCAGTATTTAATAG |
| *GmBTB/POZ* | F: actcttgaccatggtagatctgGCCGATGTCGTCCTCGTTC |
| *-6-1302* | R: aagttcttctcctttactagtAAATGCGCCTGCAGTATTTAATAG |
| *GmBTB/POZ* | F: actcttgaccatggtagatctgAATTGGGATAAAGCTATTTCTAACTATGC |
| *-7-1302* | R: aagttcttctcctttactagtAAATGCGCCTGCAGTATTTAATAG |

**Primers for transient expression assay**

| Primer name | Primers (5'-3') |
|---|---|
| *GmLHP1-BD* | F: GAATTCATGAAGGGTGGTGGTGGGA |
| | R: GTCGACTCACAATGTAGGGCTGTAGCGA |

**Primers for ChIP assays**

| Primer name | Primers (5'-3') |
|---|---|
| *GmWRKY40-a* | F: ATGATTGCCTGCCCTCG |
| | R: CCATCAAGTCGGGATAGAAGT |
| *GmWRKY40-b* | F: AGACAGACTTCTATCCCGACTT |
| | R: TTTTTCTTGGGGATTAGGTTT |
| *GmWRKY40-c* | F: CAGAAAACCTAATCCCCAAG |
| | R: GCTGGAAAAGGAAGGGAAT |
| *GmWRKY40-d* | F: TTCCCTTCCTTTTCCAGC |
| | R: TGAGTAACACGGAAGTAGATGC |
| *GmEF1-a* | F: AGAGAGACTTATGGCAAAGACAA |
| | R: CTTCCTTCTTCTCCCCGC |
| *GmEF1-b* | F: GATGCGGGGAGAAGAAGG |
| | R: TCACTTTTTGGTTCCTTTATCTT |
| *GmEF1-c* | F: TCGGGTCAGATGTCGGATG |
| | R: TACAGTGCGAGCGAGCGT |

**Primers for transient transcription dual-luciferase assay**

| Primer name | Primers (5'-3') |
|---|---|
| *GmWRKY40-LUC* | F: GGAAGCTTAATTTCTTGCAGACCAACTGAC |
| | R: GGGATCCCAAAGCTGGCATTCTTTATTTT |

**Primers for Promoter-GUS analysis**

| Primer name | Primers (5'-3') |
| --- | --- |
| *GmWRKY40-GUS* | F: GGAAGCTTAATTTCTTGCAGACCAACTGAC |
| | R: GGGATCCCAAAGCTGGCATTCTTTATTTT |
