# Supplementary material for: GmBTB/POZ promotes the ubiquitination and degradation of LHP1 to regulate the response of soybean to Phytophthora sojae
Source: Commun Biol. 2021 Mar 19;4:372. doi: 10.1038/s42003-021-01907-7 (PMC7979691; doi:10.1038/s42003-021-01907-7)
Supplement: Supplementary file 3 — Description of Additional Supplementary Files [file 42003_2021_1907_MOESM3_ESM.pdf]

Description of Additional Supplementary Files

File Name: Supplementary Data 1

Description: Source data underlying the graphs presented in the main figures and supplementary figures.

File Name: Supplementary Data 2

Description: All RNA-Seq analysis data on GmLHP1OE vs. wild-type soybean plants.
