# Supplementary material for: GmBTB/POZ promotes the ubiquitination and degradation of LHP1 to regulate the response of soybean to Phytophthora sojae
Source: Commun Biol. 2021 Mar 19;4:372. doi: 10.1038/s42003-021-01907-7 (PMC7979691; doi:10.1038/s42003-021-01907-7)
Supplement: Supplementary file 6 — Reporting Summary [file 42003_2021_1907_MOESM6_ESM.pdf]

nature research

Corresponding author(s): Shuzhen Zhang
Last updated by author(s): Jan 30, 2021

Reporting Summary

Nature Research wishes to improve the reproducibility of the work that we publish. This form provides structure for consistency and transparency in reporting. For further information on Nature Research policies, see our Editorial Policies and the Editorial Policy Checklist.

Statistics

For all statistical analyses, confirm that the following items are present in the figure legend, table legend, main text, or Methods section.

n/a	Confirmed	
[ ]	[x]	The exact sample size (*n*) for each experimental group/condition, given as a discrete number and unit of measurement
[ ]	[x]	A statement on whether measurements were taken from distinct samples or whether the same sample was measured repeatedly
[ ]	[x]	The statistical test(s) used AND whether they are one- or two-sided<br>*Only common tests should be described solely by name; describe more complex techniques in the Methods section.*
[x]	[ ]	A description of all covariates tested
[ ]	[x]	A description of any assumptions or corrections, such as tests of normality and adjustment for multiple comparisons
[ ]	[x]	A full description of the statistical parameters including central tendency (e.g. means) or other basic estimates (e.g. regression coefficient) AND variation (e.g. standard deviation) or associated estimates of uncertainty (e.g. confidence intervals)
[x]	[ ]	For null hypothesis testing, the test statistic (e.g. *F*, *t*, *r*) with confidence intervals, effect sizes, degrees of freedom and *P* value noted<br>*Give P values as exact values whenever suitable.*
[x]	[ ]	For Bayesian analysis, information on the choice of priors and Markov chain Monte Carlo settings
[x]	[ ]	For hierarchical and complex designs, identification of the appropriate level for tests and full reporting of outcomes
[x]	[ ]	Estimates of effect sizes (e.g. Cohen's *d*, Pearson's *r*), indicating how they were calculated

Our web collection on statistics for biologists contains articles on many of the points above.

Software and code

Policy information about availability of computer code

Data collection	Genes sequences, involved in this study, were obtained from NCBI database (https://www.ncbi.nlm.nih.gov/) and Phytozome (https://phytozome.jgi.doe.gov/). NLS Mapper software (http: //nls - mapper.iab.keio.ac.jp) was used to analyze the nuclear localization signal regions of the protein.
Data analysis	In this study, ImageJ software (http://imagej.nih.gov/ij/index.html) was used for band density analysis.

For manuscripts utilizing custom algorithms or software that are central to the research but not yet described in published literature, software must be made available to editors and reviewers. We strongly encourage code deposition in a community repository (e.g. GitHub). See the Nature Research guidelines for submitting code & software for further information.

Data

Policy information about availability of data

All manuscripts must include a data availability statement. This statement should provide the following information, where applicable:
- Accession codes, unique identifiers, or web links for publicly available datasets
- A list of figures that have associated raw data
- A description of any restrictions on data availability

We declare that all data supporting the findings of this study are available within the paper and its supplementary information.

Field-specific reporting

Please select the one below that is the best fit for your research. If you are not sure, read the appropriate sections before making your selection.

☒ Life sciences ☐ Behavioural & social sciences ☐ Ecological, evolutionary & environmental sciences

For a reference copy of the document with all sections, see nature.com/documents/nr-reporting-summary-flat.pdf

Life sciences study design

All studies must disclose on these points even when the disclosure is negative.

Sample size	In this study, sample sizes of at least six were chosen, which represents detection of a 20% difference in the mean among groups with a probablity of greater than 95% (P<0.05).
Data exclusions	In this study, no data were excluded from the analyses.
Replication	In this study, we confirmed that all attempts at replication were successful.
Randomization	In this study, the samples from experimental groups were collected by random sampling methods.
Blinding	For data collection and analysis, the investigators were blinded to group allocation.

Reporting for specific materials, systems and methods

We require information from authors about some types of materials, experimental systems and methods used in many studies. Here, indicate whether each material, system or method listed is relevant to your study. If you are not sure if a list item applies to your research, read the appropriate section before selecting a response.

Materials & experimental systems

n/a	Involved in the study	
☐	☒	Antibodies
☒	☐	Eukaryotic cell lines
☒	☐	Palaeontology and archaeology
☒	☐	Animals and other organisms
☒	☐	Human research participants
☒	☐	Clinical data
☒	☐	Dual use research of concern

Methods

n/a	Involved in the study	
☒	☐	ChIP-seq
☒	☐	Flow cytometry
☒	☐	MRI-based neuroimaging

Antibodies

Antibodies used	anti-GST mouse antibody (Abmart, code number M20007S); anti-His mouse antibody (Abmart, code number M20001S); anti-Flag-Tag Mouse mAb (Agarose Conjugated) (Abmart, code number M20018S); anti-Flag mouse antibody (Abmart, code number M20008M); anti-Ubi rabbit antibody (Abcam code number ab19169); anti-Myc mouse antibody antibody (Abmart code number M20002M); anti-plant actin mouse antibody (Abbkine code number A01050)
Validation	The peroxidase-conjugated affinipure goat anti-mouse/rabbit IgG as the secondary antibody.
